# Tracking hot electrons in single-atom alloy with small-molecule probes

**DOI:** 10.1093/nsr/nwaf261

**Published:** 2025-07-08

**Authors:** Zhirun Xie, Hao Wu, Yun Hau Ng

**Affiliations:** Center for Renewable Energy and Storage Technologies (CREST), Physical Science and Engineering (PSE) Division, King Abdullah University of Science and Technology (KAUST), Saudi Arabia; Clean Energy Research Platform (CERP), Physical Science and Engineering (PSE) Division, King Abdullah University of Science and Technology (KAUST), Saudi Arabia; Macau Institute of Materials Science and Engineering (MIMSE), Faculty of Innovation Engineering, Macau University of Science and Technology, China; Center for Renewable Energy and Storage Technologies (CREST), Physical Science and Engineering (PSE) Division, King Abdullah University of Science and Technology (KAUST), Saudi Arabia; Clean Energy Research Platform (CERP), Physical Science and Engineering (PSE) Division, King Abdullah University of Science and Technology (KAUST), Saudi Arabia

Upon photoexcitation, hot electrons within metallic nanostructures often demonstrate exciting phenomena when they are ‘interacting’ with the adjacent environment. These interactions, influenced by various factors, can involve internal coupling with phonons or the surrounding lattice, leading to cooled (low-energy) electrons or the formation of polarons [1,2]. When hot electrons are transferred to external molecules, they can modulate the adsorption–desorption processes and activation behaviors. Consequently, harnessing or manipulating hot electrons presents a promising approach for tuning selective photocatalysis. Direct quantification of hot electrons remains highly challenging due to their short lifetime (in the order of femtoseconds) and short travel distance (free paths in <10 nm) [3]. Ultrafast spectroscopy has significantly advanced our understanding of hot electrons, particularly in relation to the macroscopic photoelectric effect. Nonetheless, tracking the generation and transmission of hot electrons at the atomic scale, with the possibility of revealing their direct role in altering reaction mechanistic pathways, continues to be an unresolved challenge.

**Figure 1.**
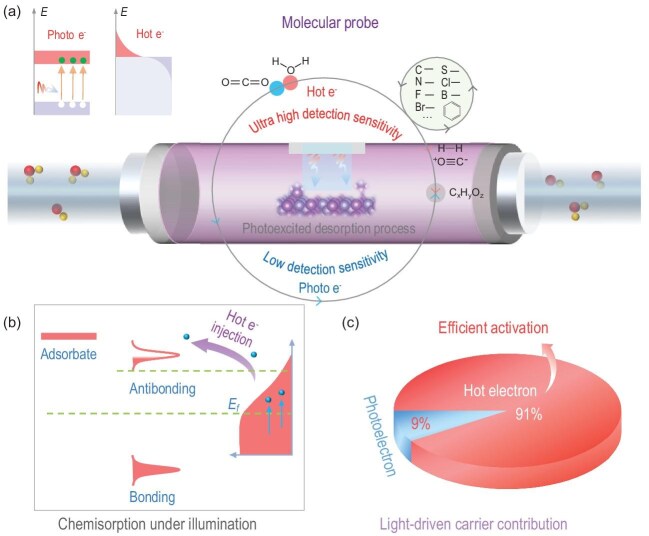
(a) Schematic drawing of the *in situ* photoexcitation desorption setup to study the atomic-scale photoelectric effect. (b) Schematic illustration of hot-electron promoted adsorption–desorption using molecular orbital perspective. (c) Quantification of contributions between hot electron and photoelectron. Reproduced with permission from [4].

Li *et al.* recently developed an *in situ* photoexcitation desorption analyzer (Fig. 1a) to address this challenge [4]. They first detected the hot-electron generation and transfer in single-atom alloys, then advanced to quantitatively identify reactive sites in photocatalytic syngas production (CO and H_2_). Li and his team synthesized FeV single-atom alloy loaded onto FeVO_4_ photocatalyst (FeV@FeVO). Under light, hot electrons are generated in the vicinity of the FeV single-atom alloys, while photoelectrons are predominantly produced in FeVO_4_.

The transfer of generated hot electrons can be facilitated when there are molecular probes adsorbed onto the catalyst surface. In this study, illuminated FeV single-atom alloys produced a substantial number of energetic hot electrons, which were subsequently transferred to CO_2_ molecules acting as adsorbate, populating the initially unoccupied antibonding orbitals (see Fig. 1b). As a result, desorption of the CO_2_ is promoted. By quantifying the amount of desorbed CO_2_ molecules, the transfer of hot electrons can be monitored effectively. Besides CO_2_, various molecular probes interact differently with hot electrons. The electrophilicity (*ω*) of these molecular probes can be estimated [5], with more electrophilic probes favoring adsorption on the single-atom or metal surface. Under illumination, the desorption and activation of more electrophilic probes increase. The electrophilicity hierarchy observed in this study is: CO > CO_2_ > C_2_H_4_ > C_3_H_6_. Conversely, nucleophilic molecules such as C_3_H_8_ and C_2_H_6_ exhibit less affinity for hot electrons, resulting in diminished activation and desorption effects. The sensitivity of hot electron detection (*χ*) was proposed by the team based on the number of active sites and the electrophilicity of the molecular probes.

Since hot electrons in single-atom alloys are generated by the electromagnetic field of light, their production is not constrained by the light's wavelength. This means that hot-electron generation is not limited to illumination with energy exceeding the photocatalyst's band gap. By using ultraviolet, visible light or near-infrared (NIR) light sources, the team can adjust the participation levels of both hot electrons and photoelectrons, as illustrated in Fig. 1c. Interestingly, under NIR illumination—where only hot electrons are produced—reactions still proceed efficiently. In single-atom configurations, hot electrons experience fewer losses due to less confinement of the light field compared to bulk materials. Consequently, hot electrons in these environments have longer lifetimes and contribute to enhanced photoactivity. The team has demonstrated that hot electrons facilitate the activation of CO_2_ and H_2_O, promoting dissociative adsorption and activation to produce syngas (CO and H_2_).

In essence, Li *et al.* developed a novel *in situ* experimental technique to track and quantify the generation and utilization of light-induced hot electrons directly at the site of reactions. This original work also distinguishes the atomic-scale photoelectric effect from its macroscopic counterpart. This study presents an innovative and effective approach for tracking and evaluating hot electrons, facilitating their application in green chemicals synthesis.
